# Vaccine strategies for the *Mtb*/HIV copandemic

**DOI:** 10.1038/s41541-020-00245-9

**Published:** 2020-10-13

**Authors:** Riti Sharan, Deepak Kaushal

**Affiliations:** Southwest National Primate Center, Texas Biomedical Research Institute, San Antonio, TX 78227 USA

**Keywords:** Diseases, Microbiology, Pathogenesis, Immunology

## Abstract

One-third of world’s population is predicted to be infected with tuberculosis (TB). The resurgence of this deadly disease has been inflamed by comorbidity with human immunodeficiency virus (HIV). The risk of TB in people living with HIV (PLWH) is 15–22 times higher than people without HIV. Development of a single vaccine to combat both diseases is an ardent but tenable ambition. Studies have focused on the induction of specific humoral and cellular immune responses against HIV-1 following recombinant BCG (rBCG) expressing HIV-1 antigens. Recent advances in the TB vaccines led to the development of promising candidates such as MTBVAC, the BCG revaccination approach, H4:IC31, H56:IC31, M72/AS01 and more recently, intravenous (IV) BCG. Modification of these vaccine candidates against TB/HIV coinfection could reveal key correlates of protection in a representative animal model. This review discusses the (i) potential TB vaccine candidates that can be exploited for use as a dual vaccine against TB/HIV copandemic (ii) progress made in the realm of TB/HIV dual vaccine candidates in small animal model, NHP model, and human clinical trials (iii) the failures and promising targets for a successful vaccine strategy while delineating the correlates of vaccine-induced protection.

## Introduction

A person living with human immunodeficiency virus (HIV) is estimated to be 20–30 times more susceptible to developing active tuberculosis (ATB)^1^. An estimated 10 million people were infected with *Mycobacterium tuberculosis* (*Mtb*) globally in 2018, with 251,000 deaths among HIV-positive people^2^. TB is endemic in poverty-stricken regions where treatment of symptoms seldom reduces the disease burden. Geographically, 87% of the world’s TB infected population had a documented HIV test result, out of which 86% were in HIV+ individuals who were on anti-retroviral therapy (ART). While there is no licensed vaccine against HIV, Mycobacterium *bovis* bacillus Calmette-Guerin (BCG) is the only licensed vaccine for TB to date^3^. The protection provided by BCG is age-dependent and variable^4–7^. An effective, preventive dual TB/HIV vaccine remains crucial to end the global pandemic. The immunization strategy together with treatment such as ART could provide long-term efficacy. Development of a combined vaccine to combat both TB and HIV is an ardent but tenable ambition. Numerous studies have focused on the induction of specific humoral and cellular immune responses against HIV-1 following recombinant BCG (rBCG) expressing HIV-1 antigens^8–13^. rBCG is an excellent vaccine vehicle that elicits the prerequisites of a successful HIV vaccine; neutralizing antibodies, stimulation of CD4^+^ and CD8^+^ T cells and a long-lasting innate and adaptive immune response^10,14,15^. On the other hand, recent advances in TB vaccines have presented promising candidates such as MTBVAC, revaccination with BCG, H4:IC31 and H56:IC31^16–19^ that have the potential to be modified for use against TB/HIV coinfection. One such candidate, MTBVAC.HIVA^2*auxo*^, a live-attenuated vaccine for HIV-1 and TB elicited polyfunctional HIV-1-specific CD8+ T cells and interferon-γ-producing *Mtb*-specific T cells^20^ (Table 1).

**Table 1:** Overview of potential vaccine candidates against TB/HIV copandemic.

Vaccine Type	Adjuvant/Boost	TB Response	HIV Response	Mouse Testing	NHP Testing	Human Trials	Outcomes
MTBVAC Live attenuated	–	Polyfunctional CD4+ central memory T cells	None	Yes	No	Phase I completed, Phase II in progress	Safe and immunogenic as BCG
BCG.HIVA^2*auxo*^ BCG vectored	MVA.HIVA Boost	IFNg + T cells	IFNg+ TNFa+ CD107a+ CD8+ T cells	Yes	No	No	Safe and immunogenic in mice
MTBVAC.HIVA^2*auxo*^ Live attenuated	MVA.HIVA Boost	Th1 type immune response	Virus-specific polyfunctional CD8+ T cells	Yes	No	No	Protective efficacy similar to MTBVAC in mice
BCG.HIV consv1&2^2auxo.int^ BCG vectored	ChAdOx1. tHIVconsv Boost	PPD-specific T cell responses	HIV-1 specific IFNg+ T cells	Yes	No	No	Well tolerated and immunogenic in mice
mc^2^6435 Recombinant attenuated	rAd5SIV Boost	No pathology, no viable TB, no TB dissemination	SIV-specific CD4+ and CD8+ T cell response	No	Yes	No	Safe and immunogenic in infant macaques
AMtb-SIV	MVA-SIV Boost	Enhanced myeloid cell responses	Control of virus replication	No	Yes	No	Improved safety than BCG, immunogenic
rBCG-SIVgag Recombinant vectored	NYVAC *gag-pol*	Boosting of IFNg-PPD response	Cellular response to SIV gag	No	Yes	No	rBCG vector suitable for anti-SIV gag response
BCG IV	–	Antigen-specific T cells	None	Yes	Yes	No	Substantial limitation of *Mtb* infection
MtbΔsigH *Mtb* mutant strain	–	iBALT, central memory CD4+, CD8+ response	None	No	Yes	No	Roadmap for identifying immune correlates of protection
MVA85A Viral vector	As a boost to BCG Prime	Ag85A- response in lung	Mono-functional Ag 85A-responses	Yes	Yes	Phase IIa and IIb	Booster mucosal delivery confers higher immunogenicity
H56 fusion protein as a booster to BCG Subunit vaccine	IC31	Prevent anti-TNF triggered LTBI reactivation	None	Yes	Yes	Phase II	H56 response distinct from BCG IFNg response
ID93 Recombinant protein antigen	GLA-SE	IFNg+TNFa+ Il-2+ CD4+T cell response	None	Yes	Yes	Phase II	Safe, elicited antibody responses
M72/AS01 Recombinant fusion protien	AS01	Polyfunctional M72-specific CD4+ T cells	M72-specific antibodies	Yes	No	Phase I/II	Safe, induced sustained responses
H4:IC31 Subunit vaccine	IC31	IFNg+TNFa+ Il-2+ CD4+ T cell response	None	Yes	No	Phase II	Potential biological effect in *Mtb* control
AERAS-402 /AD35.TB-S Fusion protein	As a booster to BCG prime	Antigen-specific CD4 and CD8 response	No viral shedding	Yes	Yes	Phase I	Well tolerated and immunogenic

Use of a biologically relevant animal model such as nonhuman primates (NHP) continues to be an integral part of the preassessment before human clinical trials of new vaccine candidates. Immunogenicity and protection studies have been conducted previously in NHPs to establish safety and efficacy of *Mycobacterium*-based HIV-1/SIV vaccines^21–24^. The rhesus macaque model was recently utilized to validate protective immunity against *Mtb* in the context of SIV coinfection^25^. Aerosol vaccination of rhesus macaques with *MtbΔsigH* prior to SIV infection was able to induce bronchus-associated lymphoid tissue (iBALT) and CD8^+^ effector memory T cells, in addition to reducing the bacterial burden, clinical manifestations, and granulomatous pathology^26^. More recently, the administration of BCG via intravenous route profoundly altered the immune response to a subsequent *Mtb* challenge, that can have important implications in the vaccine delivery^27^.

Despite these advances, the induction of sustained protective immunity against *Mtb* in the presence of HIV-associated immune activation remains one of the prominent challenges. Though ART is a vital component in managing HIV, it only partially restores the loss of CD4+ T cell upon HIV infection^28,29^. It is unable to reverse the impact of HIV on Mtb-antigen presentation by dendritic cells^30^, impairment of B cell and antibody function, all of which play a significant role in immunity to TB^31,32^. The benefit of ART is significantly dependent on the CD4 counts in HIV patients and on concurrent TB therapy in coinfected patients^33^. While ART can control viral replication in both the periphery and the alveolus, it fails to prevent SIV-induced reactivation of latent *Mtb* infection into TB disease in macaques^34^.

**Fig. 1:**
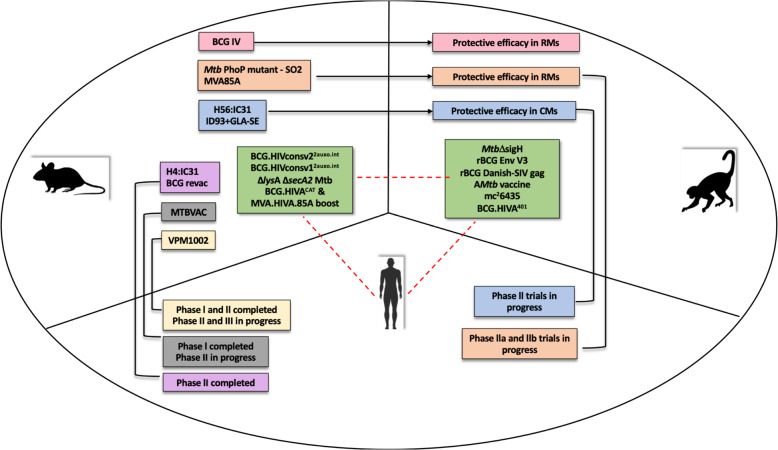
Potential vaccine candidates for TB/HIV copandemic. The figure illustrates the preclinical and clinical development of existing TB and TB/HIV vaccine candidates including small animal (mouse), NHP model, and human clinical trials.

Investigating the immune mechanisms involved in *Mtb* control in the face of HIV coinfection is key to gaining new insights into potential candidates. Recently, it has been demonstrated that CD4+ T cell-independent factors are responsible for virus-induced reactivation of latent tuberculosis infection (LTBI)^35^. These findings have paved the way for further exploration of CD8^+^ T cells and B cells in prevention of reactivation. This review focuses on the advancements made in the realm of dual vaccine candidates against *Mtb* and HIV in recent years and brings forth both, the failures and promising targets for a successful vaccine strategy (Fig. 1).

## Mouse model in TB/HIV vaccines

**Fig. 2:**
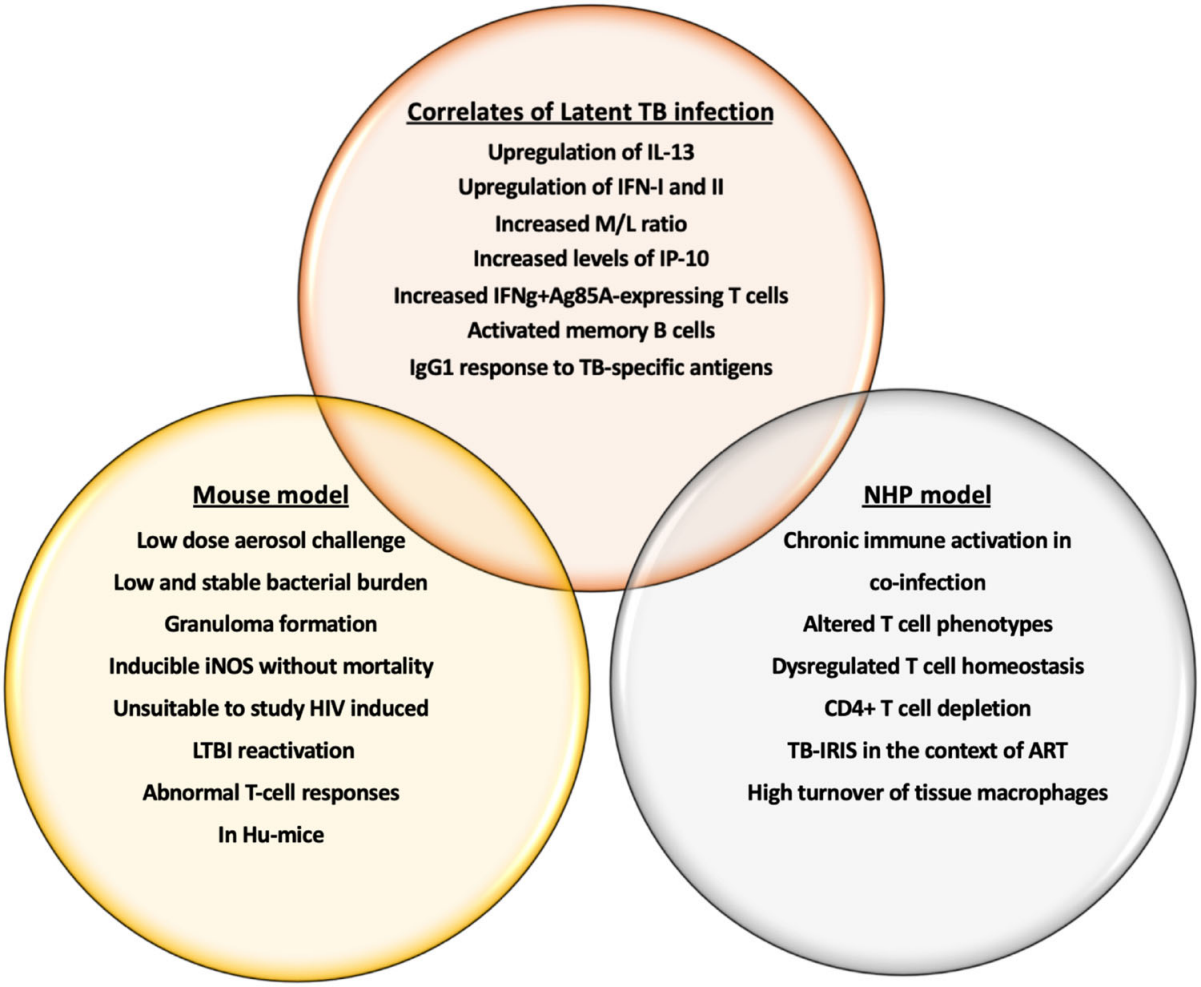
LTBI correlates in mouse and NHP model of TB/HIV coinfection. The figure outlines the immune correlates of latent TB infection that can be utilized as biomarkers in vaccine design against TB/HIV copandemic to prevent infection and reactivation. It also compares and contrast the mouse and NHP model in terms of mimicking human infection and immune response in TB/HIV coinfection.

A wide array of possibilities such as the use of live-attenuated bacteria, integrative vectors, and protein subunit adjuvant have been placed in the pipeline for preclinical testing^36^ of vaccines to prevent HIV-related tuberculosis. The possibility of a joint TB-HIV vaccine design is based on a (i) mycobacterial-based live vaccine vehicle, (ii) induction of potent T_H_1 immune responses, and (iii) antibiotic-free plasmid selection system. Nusbaum et al. have performed pioneering work in developing a humanized mouse model to understand the HIV-1 led disruption of pulmonary TB containment^37^. Coinfection of the bone marrow, liver, thymus (BLT) humanized mouse (HuMice) with TB and HIV-1 exacerbated the proinflammatory response to pulmonary *Mtb*. Interestingly, the inflammatory cytokine signature was HIV-1 induced that led to poorly formed granulomas and disease dissemination^37^. The model provided a better understanding of the conflicting perspective of immune activation and immune suppression in coinfected human subjects. Indeed, the mouse model is widely used for preclinical testing of safety and efficacy of novel vaccine candidates. Some models have been able to demonstrate key LTBI correlates such as low-dose aerosol challenge^38^, low and stable bacterial burden, formation of granulomas and higher expression of inducible nitric oxide synthase (iNOS) without mortality^39,40^. However, mimicking all aspects of human latent TB infection (LTBI) in mice remains a challenge (Fig. 2).

Among the promising candidates that have been successfully tested in the mouse model and are now in the human trials is MTBVAC^41^. After demonstrating improved efficacy and immunity relative to BCG in newborn mice^42^, it has recently been used as a vector for the construction of recombinant MTBVAC.HIVA^2*auxo*^ ^20^. The vaccine was safer than BCG in severe combined immunodeficiency (SCID) mice and successfully induced immune responses to both HIV-1 and *Mtb*. The vaccine candidate induced an efficacy similar to the parent MTBVAC strain. When adjuvanted with modified vaccinia virus Ankara (MVA), it induced pathogen-specific IFNγ-producing T cell responses, polyfunctional HIV-1-specific IFNγ producing CD8^+^ T cells, TNFα and CD107a in mice. The study outlined the use of a mycobacterial-based vaccine vector. 2^nd^-generation conserved-region immunogens aimed to induce T-cell responses specifically against the conserved regions of HIV-1 proteome were expressed in novel BCG-vectored vaccine candidates, BCG.HIVconsv1^2auxo.int^ and BCG.HIVconsv2^2auxo.int^. These candidates were well tolerated in adult BALB/c mice and induced HIV-1-specific T cell responses in addition to improving the mycobacterial vaccine stability and immunogenicity^43^. Murine studies to highlight the host-mycobacterial interactions using rBCG demonstrated a greater antigen-specific response in splenocytes from granulocyte-macrophage colony-stimulating factor (GM-CSF), IL-2 and IFNγ -secreting BCG vaccinated mice compared to mice injected with BCG lacking cytokines^44^. The potentiation of the immune-stimulatory properties of BCG through secretion of mammalian cytokines confirmed that a large fraction of murine cytokines, such as IL-2, IL-4, IL-6, IFNγ could be produced and secreted in their active forms by BCG. The changes in cellular response appeared to be quantitative and macrophage-driven, making the inclusion of cytokine with BCG an attractive candidate for TB/HIV vaccine. BCG Δ*ureC::hly* (rBCG, VPM1002), is protective against *Mtb* in mice through the membrane-perforating listeriolysin of *Listeria monocytogenes*^45^. rBCG was able to induce a higher absolute number and proportions of antigen-specific central memory CD4+ T cells than BCG that were maintained after clearance of rBCG in mice. Targeting the expansion of central memory CD4+ T cells when designing a coinfection vaccine could lead to improved, long-term protection. Dey et al. proposed the development of BCG-vectored STING agonists as a tuberculosis vaccine strategy^46^. They created a rBCG (BCG-disA-OE) that releases high levels of STING agonist by overexpression of endogenous mycobacterial deadenylate cyclase gene. They hypothesized that the overproduction of STING could result in enhanced protective efficacy of BCG against pulmonary and extrapulmonary TB. Enhancing innate immune activation along with enduring antigen-specific Th1 responses and Th17 responses via STING -activating adjuvants could be desirable in TB/HIV coinfection vaccine design.

While a number of studies report characterization of successful *Mtb*/HIV coinfection mouse model^37,47–49^, it remains largely unsuitable to study the HIV coinfection induced reactivation of a latent TB infection (LTBI). In addition, the mouse model is limited in terms of reliability as an efficient evaluation system for long-term protective immune responses. Previous attempts of establishing a latent-relapse humanized TB mouse model led to varied latency period, levels of relapse and higher than predicted bacterial burden during latency^50–52^.

## Nonhuman primate model for TB/HIV vaccine evaluation

Macaques are generally considered to be a highly representative model for modeling TB due to their physiological, pathological, and immunological similarity to humans^53–56^. In addition, the NHP model is considered the most reliable and translatable model to test for preclinical trials of vaccine candidates^57–59^ due to its ability to get coinfected with both TB and SIV. As such they may also be highly suitable for testing new TB/HIV vaccine candidates that can protect against HIV-related TB. Some of the earlier work in the field focused on development of a pediatric combination HIV-TB vaccine that was predicted to be safe, have a comparable immunogenicity to BCG and could be modified to co-express HIV genes^60^. *Mtb* strain, mc^2^6435, attenuated in genes critical in replication and immune evasion, carrying SIV Gag expression plasmid was observed to be safe in SIV-infected and non-SIV-infected infant rhesus macaques^60^. The development of mc^2^6435 as a TB vaccine candidate for HIV-infected population was based on earlier attempts to develop strains mc^2^6020 and mc^2^6030 as potential candidates in cynomolgus macaques. Though these strains were well tolerated in primates, they provided only partial protection against *Mtb* challenge^61^. Recently, the same group performed vaccination of infant rhesus macaques with a pediatric combination vaccine containing an auxotroph *Mtb* strain co-expressing HIV antigens, *AMtb*, that demonstrated enhanced myeloid cell responses and a possible attenuation of immune activation^62^. This vaccine strain was conceptually similar to the MTBVAC but was limited in replication within the mammalian host. The candidate vaccine was able to enhance the functional responses of monocytes/macrophages after a single immunization at birth in addition to inducing CD4+, CD8+ T cells, and B cells^62^.

**Fig. 3:**
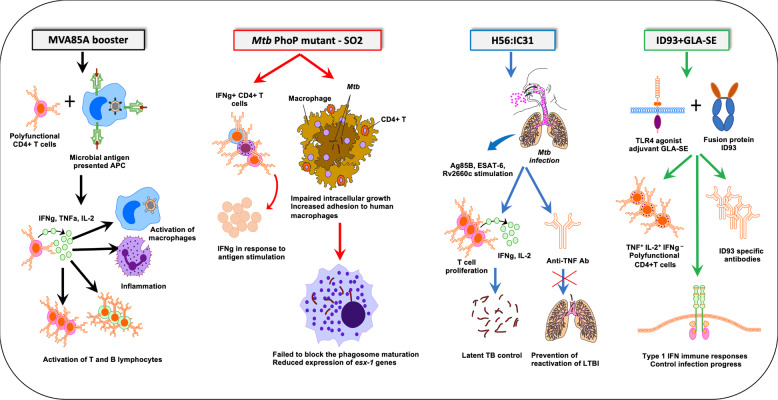
Mechanism of action of the key vaccine candidates with a potential to combat TB/HIV copandemic. Aerosol delivery of a leading TB vaccine candidate, modified vaccinia virus Ankara expressing antigen 85 A (MVA85A) in rhesus macaques produced a higher immune response compared to intradermal injection highlighting an immunization strategy that limits systemic immunity. Novel TB vaccine candidate, pho P mutant SO2 was unable to induce apoptotic events during lung infection *in vivo*. H56 fusion protein (Ag85B-ESAT6-Rv2660c) has been developed as a BCG booster in cynomolgus macaques. In addition to delaying the clinical disease manifestation post *Mtb* infection, H56 booster was able to prevent anti-TNF triggered reactivation of latent TB infection. As observed with H56:IC31, ID93/GLA-SE elicited a significant T_H_1 immune response, comprising of multifunctional IFN-γ, TNF-α, and IL-2 CD4^+^ T cells. The induction of a dominant T_H_1 response was associated with reduced TB burden in cynomolgus macaques and MDR-TB control in the lungs of vaccinated mice.

Vaccination of rhesus macaques with BCG vectors expressing SIV-*gag* elicited baseline humoral and cellular immune responses to *Mtb*^11^. In addition to the mycobacterial response, the vaccinated primates also elicited a strong response to SIV *gag* and this response was independent of the baseline mycobacterial immunity^11^. Recent advances in the preclinical testing of TB vaccines in NHPs brings forth the potential of these candidates in TB control in coinfected cohorts. Intravenous administration of BCG induced a significantly higher antigen-responsive CD4 and CD8 T cells responses in extrapulmonary organs such as spleen and lymph nodes^27^. The model provides a critical tool in defining the underlying mechanisms of vaccine-induced protection in TB (Table 2). Since the route of delivery of the vaccine plays a significant role in eliciting both systemic and tissue-specific immunity, optimization of this variable is key to limiting bacterial replication in HIV coinfected individuals. A recent study reported substantial limitation of *Mtb* infection following intravenous (IV) administration of BCG in the highly susceptible rhesus macaque model^27^. IV BCG immunization resulted in a significant increase in antigen-specific T cells and a marked protection from *Mtb* challenge. The study is a paradigm shift towards alternative routes to improve protective capacity of vaccine platforms. While this model may be useful to identify correlates of protection, intravenous vaccinations are unlikely to be utilized in children or adults due to considerable safety concerns. Aerosol delivery of the vaccine directly to the respiratory mucosa has been considered a relatively effective route of vaccination in TB^63^. Aerosol delivery of a leading TB vaccine candidate, modified vaccinia virus Ankara expressing antigen 85A (MVA85A) in rhesus macaques produced a higher immune response compared to intradermal injection highlighting an immunization strategy that limits systemic immunity^63^. Utilizing this route of delivery enabled the induction of antigen-specific polyfunctional CD4 and CD8 T cells, expressing interferon gamma (IFN-γ), tumor necrosis factor alpha (TNF-α), and interleukin 2 (IL-2), all of which are associated with TB and HIV coinfection, thus potentiating the use of this vaccine in the coinfected cohort (Fig. 3). Combination of BCG with MVA.85A prime boost regime in rhesus macaques elicited a significant increase in the protective efficacy^64^. BCG/MVA vaccinated macaques showed significantly reduced lung lesions, chest X-ray scores, and systemic inflammation leading to better TB control. If successful in the human clinical trials, it has the potential to be used as a booster to BCG in HIV coinfected individuals. The recent WHO guidelines recommend BCG vaccination to HIV-infected individuals, including children, who are receiving ART and are clinically stable^65^. However, a sustained immunity provided through this practice could be questionable in the long run. Dijkman et al., assessed pulmonary mucosal delivery of BCG in rhesus macaques^66^. This strategy not only reduced local TB disease where standard intradermal injection failed but also prevented repeated limiting-dose *Mtb* challenge by producing polyfunctional Th17 cells, IL-10, and IgA as correlates of protective immunity. If taking this approach in a coinfection vaccine, it will be imperative to ascertain the sustained immunity to reinfections and to interrogate the contralateral strategy to assess whether the protective phenotype against endobronchial instillation extends beyond the vaccine-targeted lung lobe.

**Table 2:** Immune correlates of interest in NHP model of TB/HIV coinfection.

Immune correlate	Significance	NHP model
sCD14	Marker of microbial translocation in SIV, prediction of immune activation in TB/HIV coinfection	Rhesus macaque—SIV model
CRP	Soluble markers of inflammation, monocyte turnover, fibrosis predict progression to AIDS and death	Rhesus macaque—TB and SHIV/SIV model
IL-6		
IL-8		
IP-10	Independent marker of rapid disease onset in HIV	Rhesus/Cynomolgus macaque—SIV, TB model
HLA-DR	T-cell activation marker, predisposes to SIV and TB progression, shorter survival, mortality upon cART initiation	
CD38		
IL-2Rα	Marker of T-helper function	Sooty mangabeys—SIV model Rhesus macaque—TB model
CD45RO	Marker for primed/memory T cells	Rhesus macaque—SIV model
ESAT-6/CFP-10 specific CD4+ T cells	Key immune cell type required to contain *Mtb* within granulomas	Rhesus, Mauritian cynomolgus macaques—TB/SIV model
IFNg+IL-2+TNFa+ T cells	Marker of disease control and protection against depletion of mucosal CD4+ T cells	Rhesus macaque—TB and SIV model
CD4+CD25+CD127- Tregs	Marker to differentiate intestinal Tregs from lymphoid tissue Tregs in TB and SIV infections	Cynomolgus macaque—TB model Rhesus macaque—SIV model
Monocyte:Lymphocyte ratio	Marker to distinguish between ATB, LTBI; prediction of TB risk in infants	Rhesus, Chinese and Mauritian Cynomolgus macaque—TB model
Granzyme B	Cytotoxic T -lymphocyte mediated defence against intracellular pathogens	Rhesus, Cynomolgus macaque—TB model Rhesus macaque—SIV model
CD8+memory T cells	Suppression of viremia by rapid expansion and localization within B-cell follicles in SIV; immunity against *Mtb* reinfection	Rhesus macaque—TB and SIV model
Th17 response	Significantly reduced in SIV, can reduce *Mtb* control in lungs	Rhesus macaque—SIV model

H56 fusion protein (Ag85B-ESAT6-Rv2660c) has been developed as a BCG booster in cynomolgus macaques. Out of the 4 adjuvants (CAF01, CAF04, CAF05, and IC31) tested, H56 given in IC31 promoted the best protection (Fig. 3). In addition to delaying the clinical disease manifestation post *Mtb* infection, H56 booster was able to prevent anti-TNF triggered reactivation of latent TB infection^67^. The same study explored the importance of adjuvants by comparing H56 in three liposomal adjuvants, cationic adjuvant formulation 01 (CAF01), CAF04, and CAF05. While H56 boosted BCG-immunity in all four adjuvants tested, CAF04/05 was able to induce higher IFN-γ responses compared to CAF01. Since no correlation was observed between the IFN-γ response and protection, it is important to focus on the choice of adjuvant when optimizing TB/HIV coinfection vaccine. Contrary to the correlation of BCG/MVA85A-induced IFN-γ with protection in macaques^64^, H56 responses post *Mtb* challenge were distinct from BCG-induced systemic IFN-γ response^67^. Despite the modest levels of IFN-γ, H56 booster with IC31 adjuvant elicited a high protection level due to a response that was dominated by central memory-like T cells that produced TNF-α and IL-2^67–69^. Since an improved HIV control is associated with the production of two or more different cytokines by multifunctional CD4^+^ T cells^70^, this vaccine candidate could elicit durable T-cell responses against both *Mtb* and HIV in substantial magnitude. ID93, a candidate TB vaccine antigen formulated in a synthetic nanoemulsion adjuvant, GLA-SE has been tested as an adjunct to antibiotic treatment against TB in cynomolgus macaques for safety and efficacy^71^ (Fig. 3). GLA-SE is a synthetic TLR-4 agonist that adds an innate signal and potent Th1-inducing properties to ID93. GLA-SE was developed originally as a synthetic mono-phosphoryl lipid (MPL) which was then formulated in a stable oil-in-water emulsion^72^. A combination of existing first-line antibiotics rifampicin, isoniazid (INH), and ID93/GLA-SE could resolve *Mtb* infection in 40% of the treated macaques. Interestingly, the treated macaques responded with a significant reduction in *Mtb* bacterial numbers, negative chest radiograph, and healthy organs as determined by pathological findings^71^. As observed with H56:IC31, ID93/GLA-SE elicited a significant T_H_1 immune response, comprising of multifunctional IFN-γ, TNF-α, and IL-2 CD4^+^ T cells. The induction of a dominant T_H_1 response was associated with reduced TB burden in cynomolgus macaques and MDR-TB control in the lungs of vaccinated mice^72^. Since T_H_1 type response is immune-protective as observed in the HIV controllers, ID93/GLA-SE has the potential to prevent HIV progression in TB/HIV coinfected cohort^73,74^. These studies emphasize the valuable role of NHP model in the preclinical selection of TB/HIV vaccine candidate based on relevant biological, clinical and pathological read-outs. Recombinant BCG vaccine, AERAS-422, has been shown to induce a strong and persistent CD8+ T cell response in mice^75^. Immunogenicity testing in Chinese rhesus macaques elicited considerably higher CD4 responses and Ag85B -specific CD8 responses compared to the parent BCG^76^. However, in the first-in-human phase I trial, 2 of the 8 volunteers administered the higher dose of AERAS-422 developed Varicella zoster virus reactivations 2-3 months post-vaccination^75^. While the hypothesis for this event could be effected from overexpression of *Mtb* antigens or an imbalance of type I vs. type II IFN responses, it underscores the lack of replication or appropriate pathology in NHPs compared to humans.

In addition to the immunological similarities with humans, the NHP model offers inter-individual differences due to the genetic variation in their populations. While this presents hindrance in terms of a uniform response, it mimics the variability presented in the human population; key to development of a successful human vaccine. The advantages offered by the premier NHP model surpasses those of small animal model in terms of established integrity and reliability. The ability to test vaccine candidates in NHPs coinfected with TB and HIV offers in-depth analysis of the early events of coinfection, accurate immunological data on different phases of both the pathogens, and detailed characterization of pathology.

## Prospective HIV-based vaccine to combat HIV-related TB

HIV increases the risk of LTBI progression to ATB substantially. One of the proposed strategies to control HIV-related TB is to design an effective HIV vaccine^36^. HIV-infected individuals exhibit a significantly lower number and function of *Mtb*-specific CD4^+^ T cells in the blood and airways^77,78^. In addition, protective TB functions including T cell effector functions, long-term memory, and tissue homing potential of the immune cells are impacted by HIV^79,80^. Indeed, impaired CD4^+^ T cell immunity due to HIV coinfection in individuals with LTBI is one of the underlying causes of TB reactivation^81^. There is a preferential depletion of *Mtb*-specific IFN-γ^+^IL-2^-^TNF-α^+^ CD4^+^ T cells by HIV in these individuals with an impaired proliferative capacity of *Mtb*-specific CD4^+^ T cells^81^. Recently, mechanisms independent of CD4^+^ T cell depletion have been shown to play a pivotal role in SIV-induced LTBI reactivation in macaques, including chronic immune activation^35^, expanded B-cell follicles and CD8+ T cell proliferation^82^. In the light of these findings, it is important to utilize the NHP model to (i) better understand the impact of a vaccine-induced immunity on immune activation, (ii) better control of the coinfection, (iii) impact on the pre-existing immune responses to childhood vaccination. While designing a prospective vaccine to combat HIV-related TB, it is important to target the period of increased vulnerability of TB disease progression. The timing of vaccine administration is critical to preclude the possibility of immunosuppression post-HIV acquisition. Hence, administration of the vaccine at a younger age, prior to HIV infection, or post HIV infection but prior to ART could prove beneficial. The World Health Organization (WHO) recommends ART administration to all HIV-infected individuals^83^. While early ART is able to reduce the TB incidence in TB high, low- and moderate—burden settings, it will be crucial to evaluate the impact of ART on the vaccine candidate in this cohort^84,85^.

## TB/HIV vaccine candidates in human trials

Several TB/HIV vaccine candidates have been tested in human clinical trials over the past years^86–89^. As in the case of NHPs, the earlier work in humans focused on the development of a dual neonate vaccine platform. In this realm, one of the candidates against HIV- 1 and TB consisted of BCG.HIVA at birth followed by a booster with MVA.HIVA.85A^90^. The underlying idea was to induce immune responses against both these pathogens soon after birth that could then be maintained with boosts all throughout the life. BCG.HIVA^222^ was engineered by vectoring a lysine auxotroph of the Pasteur strain of BCG that delivered chimeric protein HIVA^91^. Yet another study highlighted the TB vaccine candidate MVA85A, a modified vaccinia virus Ankara expressing antigen 85A for safety and immunogenicity in adults infected with HIV-1^92^. This vaccine candidate was found to be safe and immunogenic in adults with HIV-1 infection but failed to demonstrate an effective immune response to *Mtb*. However, this study was underpowered to detect a sufficient vaccine-induced immune response. Recent studies in newborns of HIV-infected mothers revealed acceptable safety and reactogenicity when administered MVA85A followed by BCG vaccine boost at age 8 weeks^93^. MVA85A was able to induce a modest yet independent *Mtb*-antigen-specific immune response earlier that did not have an impact on the BCG-induced immunity later. The potential of this weak IFNγ+ Ag85A-specific T cell response before BCG vaccination to protect against TB is still questionable. However, the clinical development of this strategy could be feasible, given the non-interference of the MVA with the BCG response and vice versa. Alternating routes of administration of MVA85A via aerosol and intradermal vaccination in a phase 1 human randomized clinical trial was conducted to test the hypothesis that this may alleviate the Ag85A insertion induced immune response^94^. Administering the virus-based TB vaccine via an inhaler was well tolerated, however, vaccinating the primary dose via an injection had transient but significant respiratory adverse events. Administration of TB vaccine directly to the lungs in HIV- positive cohort could stimulate higher protective immune responses in the lungs, the primary port of entry of TB in the human body.

Safety and efficacy of the candidate TB vaccine, M72/AS01, a protein subunit vaccine showed promising results in the safety and immunogenicity studies performed in adults treated for HIV infection by ART^95^. The vaccine was well tolerated and it successfully induced persistent and polyfunctional M72-specific CD4^+^ T cell responses with the dominant populations being CD40L^+^IL-2^+^TNFα^+^, CD40L^+^IL-2^+^, and CD40L^+^IL-2^+^TNFα^+^IFNγ^+^ T cells. The vaccine could induce M72-specific antibodies irrespective of ART status in HIV-positive individuals. However, the antibody response was more persistent in the ART-stable than in ART-naïve cohort indicating that ART had a positive impact on the anti-M72 IgG responses. The safety and immunogenicity of AERAS-402/AD35.TB-S was evaluated in populations that were either BCG vaccinated or BCG-naïve populations in United States^96^ and South Africa^97^ but were essentially HIV-uninfected. A follow up of this candidate was performed in a study that included testing its safety and immunogenicity in BCG-vaccinated, HIV-infected individuals with CD4 counts > 350 cells/mm^3,87^. This candidate was safe and was well tolerated by the recipients. It was able to induce adaptive, including CD4+ T cell and CD8+ T cell responses and antibody immune responses to the vaccine antigens, Ag85A and Ag85B. ID93 + GLA-SE, a chimeric fusion protein TB vaccine candidate was well tolerated without any significant vaccine-related adverse effects in a randomized, double blind, placebo-controlled phase 1 trial in HIV-negative, BCG-vaccinated cohort^98^. A peak of vaccine-induced, durable, antigen-specific IgG and T_H_1 responses was observed after 2 administrations. The vaccinated cohort showed higher T-effector profiles and differentiation compared to unvaccinated controls. Further studies in HIV-infected and with a larger sample size could prove valuable in controlling *Mtb* infection in the highly endemic regions.

BCG revaccination to prevent TB incidence in *Mtb* naïve population has been tested in a phase II, randomized, placebo controlled, partially-blinded human clinical trial^18^. The same study tested the safety and efficacy of candidate subunit vaccine, H4:IC31. While the H4:IC31 showed a modest 30.5% efficacy against sustained QuantiFERON-TB Gold In-Tube (QFT), BCG revaccination exhibited a 45.4% efficacy warranting further clinical evaluation. The vaccine was able to control bacterial replication and mediated clearance by mobilizing antigen to the lymphoid tissues. These recent findings have provided ground to rethink BCG revaccination perhaps even in HIV-positive individuals. MTBVAC, a live-attenuated TB vaccine candidate conserves most of the T-cell epitopes deleted in BCG such as ESAT-6 and CFP-10 of the RD1 region^41^. The conservation of the genetic regions coding for the important immunodominant antigens is expected to induce more specific and durable immune responses in humans. Phase 1 human clinical trials of MTBVAC demonstrated safety and tolerability profile similar to BCG^41^. While MTBVAC was as immunogenic as BCG, it was able to elicit a higher frequency of multifunctional CD4^+^ central memory T cell response. There was a significant CFP-10 response in humans up to 6 months post-vaccination^1,99^ indicating that people with latent TB infection could be more protected to a secondary *Mtb* infection, since latent TB patients are reactive to ESAT-6 and CFP-10 stimulation. Future studies could be designed to include HIV-positive cohorts to study the efficacy of this vaccine candidate in inducing sustained TB specific immune responses in a coinfection setting.

In the recent past, the potential of BCG to induce non-specific cross-protection against pathogens that may be unrelated to the target disease has been explored. A study by Covian et al., demonstrated improved innate immune response against Candida albicans and Staphylococcus aureus upon BCG vaccination in neonates^100^. Monocytes and NK cells contributed to this non-specific protection via mechanisms independent of memory T or B cells. This phenomenon has been termed as “trained immunity”. More recently, the non-specific beneficial effects of BCG have been explored against viral infections, COVID-19^101^. The hypothesis is based on evidence from studies from around the globe that have successfully demonstrated up to 50–70% reduction in childhood and adolescent mortality by BCG^18,102^. Immunologically, BCG vaccination results in enhanced production of proinflammatory cytokines, IL-1β, TNF and IL-6 to these unrelated pathogens^103^. These changes in innate immune cell phenotypes lead to the induction of innate immune memory by BCG via epigenetically trained populations of monocytes and/or NK cells residing in bone marrow. In the context of *Mtb*/HIV coinfection vaccine, Jensen and colleagues have hypothesized that AMTB-SIV vaccine-induced trained immunity led to the enhanced CD4+ T cell activation, which in turn led to increased SIV susceptibility in infant macaques^104^. A deeper understanding of this concept is clearly required to improve the design of safer TB/HIV vaccines (Table 3).

**Table 3:** Mechanisms of impact on TB and HIV drug resistance in TB/HIV coinfection.

HIV on TB	TB on HIV	Common factors
Increases risk of TB reactivation	Cell activation	Lack of communication between TB and HIV treatment programs
Expands number of people with ATB	Excessive cytokine and chemokine production	Lack of treatment adherence
Contributes to selection for spontaneous mutations	Stimulates HIV replication	Immunocompromised state
Reinfection modifies conditions of co-existence	Accelerates progression to AIDS	Community transmission
High viral load increases prevalence of DR-TB	Low uptake of ART	Intravenous drug abuse
Timing and initiation of ART	Low rates of viral RNA suppression	Low socioeconomic status
Chronic immune activation onset during the acute phase	Increased viral replication	Late diagnosis

## Concluding remarks

It is important to direct future studies on potential vaccine candidates against TB/HIV coinfection towards reproducibility in human clinical trials. Future studies should also factor in any pre-existing immune responses to the rBCG vectors being used in the candidate vaccine. The vaccine candidate should be tested for safety in a robust preclinical animal model such as NHPs before use in humans. If the candidate is a live vector, it should be able to demonstrate a relatively low level of replication and/or clearance should be able to be measured by a readily usable soluble marker in blood or urine. When designing a dual TB/HIV vaccine candidate, it is important to consider the impact of genetic manipulations of *Mtb* on the overall immune spectrum and the impact of HIV immunogens on the metabolic burden. It is imperative to factor in the timing, magnitude and type of activated cells generated to produce an effective and robust pathogen-specific immune response. It would be ideal to have a dual *Mtb*/HIV vaccine candidate that is able to induce either (i) an immune response in *Mtb*/HIV coinfected individuals similar to the response induced in a natural *Mtb* infection in resistant individuals, (ii) a complete eradication of the pathogens or (iii) sustain the LTBI by preventing its reactivation.
